# Supplementary material for: Neuraminidase 1 promotes renal fibrosis development in male mice
Source: Nat Commun. 2023 Mar 27;14:1713. doi: 10.1038/s41467-023-37450-8 (PMC10043283; doi:10.1038/s41467-023-37450-8)
Supplement: Supplementary file 1 — Supplementary Information [file 41467_2023_37450_MOESM1_ESM.pdf]

SUPPLEMENTARY INFORMATION

Neuraminidase 1 promotes renal fibrosis development in male mice

Qian-Qian Chen [1, 2#], Kang Liu [3#], Ning Shi [4#], Gaoxiang Ma [5], Peipei Wang [6], Hua-Mei Xie [5], Si-Jia Jin [4], Ting-Ting Wei [1], Xiang-Yu Yu [1], Yi Wang [5], Jun-Yuan Zhang [1], Ping Li [1], Lian-Wen Qi [1, 4, 5*], Lei Zhang[1, 4*]

[1]State Key Laboratory of Natural Medicines, China Pharmaceutical University, Nanjing, 210009, China.

[2]School of Pharmacy, Nanjing University of Chinese Medicine, Nanjing, 210023, China

[3]Department of Nephrology, Jiangsu Province Hospital, The First Affiliated Hospital of Nanjing Medical University, Nanjing, 210029, China.

[4]School of Traditional Chinese Pharmacy, China Pharmaceutical University, Nanjing, 211198, China

[5]Clinical Metabolomics Center, China Pharmaceutical University, Nanjing, 211198, China.

[6]College of Food Science and Technology, Shanghai Ocean University, Shanghai, 201306, China

# These authors contributed equally to this work.

*To whom correspondence should be addressed:

Dr. Lei Zhang, School of Traditional Chinese Pharmacy, China Pharmaceutical University, No. 639 Longmian Road, Nanjing, 211198, China, Email: Zhanglei@cpu.edu.cn.

Dr. Lian-Wen Qi, Clinical Metabolomics Center, China Pharmaceutical University, No. 639 Longmian Road, Nanjing, 211198, China, Email: Qilw@cpu.edu.cn.

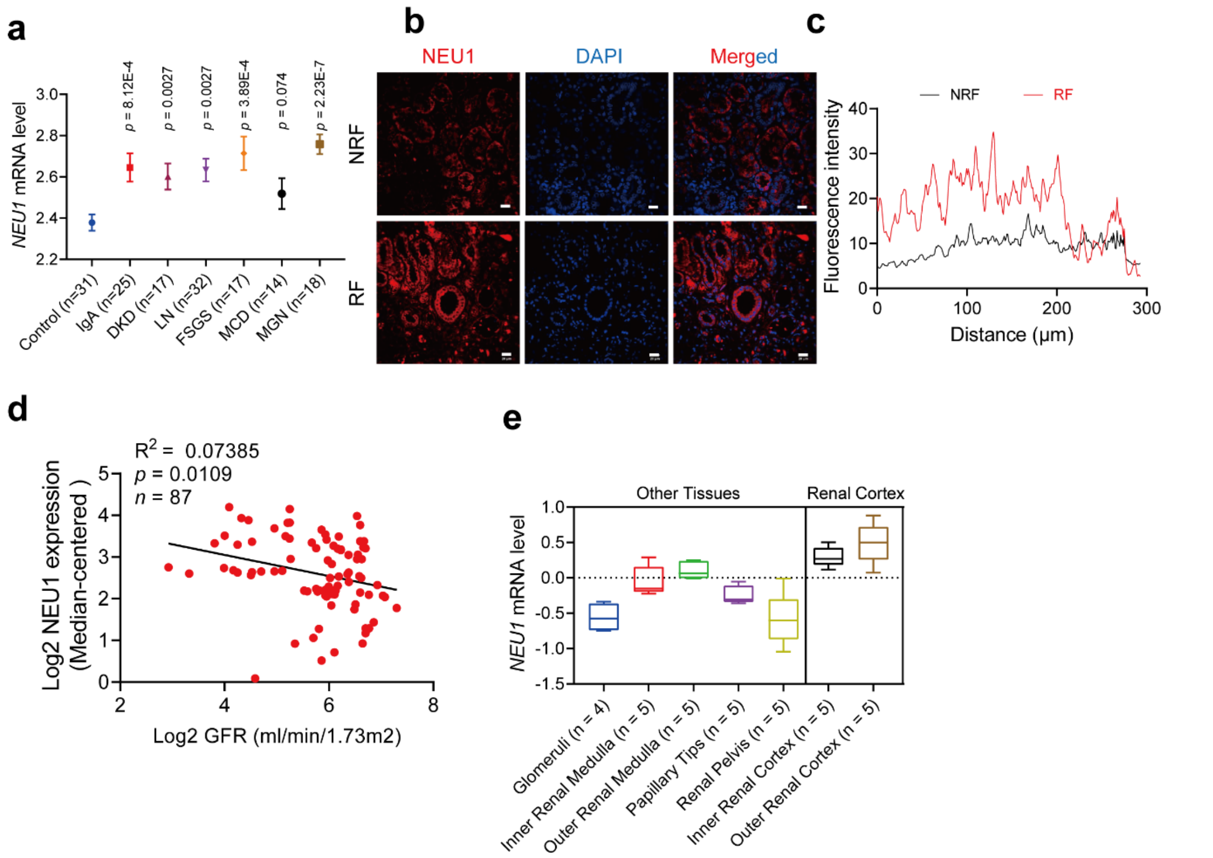

Supplementary Fig. 1 NEU1 is significantly upregulated in kidneys from patients with CKD.

a NEU1 mRNA levels in kidney tissues of control (*n* = 31) and types of chronic kidney disease (CKD) including IgA (*n* = 25), diabetic kidney disease (*n* = 17), lupus nephritis (*n* = 32), focal segmental glomerulosclerosis (*n* = 17), membranous glomerulonephritis (*n* = 18), and minimal change disease (*n* = 14). Data were presented as mean ± SEM. Unpaired two-tailed *t*-test. Data analysis from Nephroseq database (median-centered log2). ns, no significant difference. **b**, **c** Images and statistical analysis of immunofluorescence staining of NEU1 in kidney tissues of non-renal fibrosis (NRF) and renal fibrosis (RF) patients. Scale bars = 20 μm. *n* = 3 samples per group. **d** Pearson's correlation of NEU1 with GFR of control and renal fibrosis patients (Data analysis from Nephroseq database, two-tailed Pearson χ2 test). **e** Expression of NEU1 in different parts of kidney tissue. Data analysis from Nephroseq database (median-centered log2). Data are presented as box-and-whisker plots, solid line inside box indicates the median, the bottom and top of box represent first and third quartiles, and the bottom and top whisker show the minimum and maximum respectively.

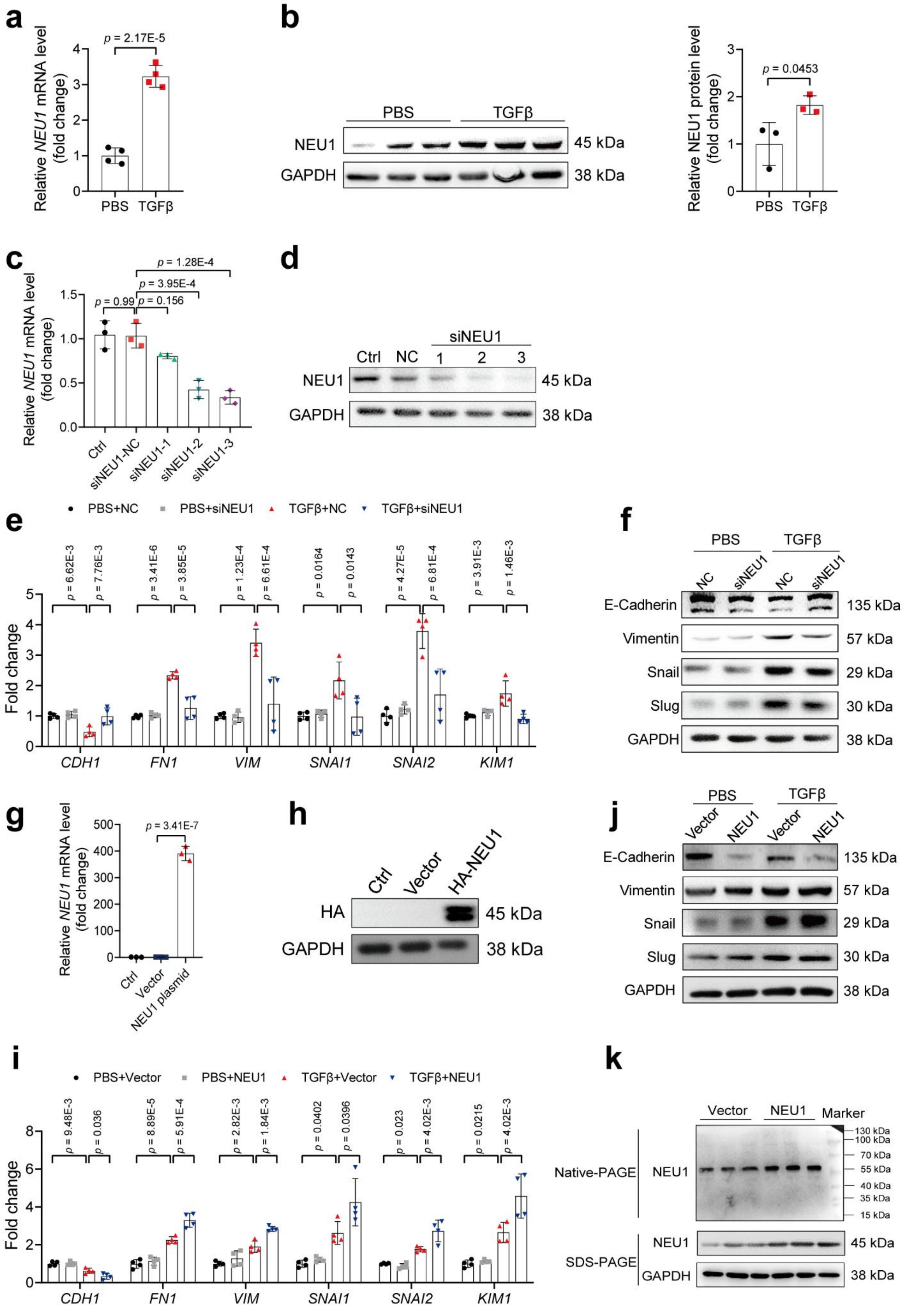
a
p = 2.17E-5
Relative NEU1 mRNA level (fold change)
PBS
TGFβ
b
PBS
TGFβ
NEU1
45 kDa
GAPDH
38 kDa
p = 0.0453
Relative NEU1 protein level (fold change)
c
p = 1.28E-4
p = 3.95E-4
p = 0.99
p = 0.156
Ctrl
siNEU1-NC
siNEU1-1
siNEU1-2
siNEU1-3
d
siNEU1
Ctrl
NC
1
2
3
e
PBS+NC
PBS+siNEU1
TGFβ+NC
TGFβ+siNEU1
p = 6.62E-3
p = 7.76E-3
p = 3.41E-6
p = 3.85E-5
p = 1.23E-4
p = 6.61E-4
p = 0.0164
p = 0.0143
p = 4.27E-5
p = 6.81E-4
p = 3.91E-3
p = 1.46E-3
Fold change
CDH1
FN1
VIM
SNAI1
SNAI2
KIM1
f
NC
siNEU1
E-Cadherin
135 kDa
Vimentin
57 kDa
Snail
29 kDa
Slug
30 kDa
g
p = 3.41E-7
Vector
NEU1 plasmid
h
HA-NEU1
HA
j
i
PBS+Vector
PBS+NEU1
TGFβ+Vector
TGFβ+NEU1
p = 9.48E-3
p = 0.036
p = 8.89E-5
p = 5.91E-4
p = 2.82E-3
p = 1.84E-3
p = 0.0402
p = 0.0396
p = 0.023
p = 4.02E-3
p = 0.0215
k
Marker
Native-PAGE
SDS-PAGE
130 kDa
100 kDa
70 kDa
55 kDa
40 kDa
35 kDa
15 kDa

Supplementary Fig. 2 NEU1 mediates the TGFβ-induced changes of TEC.

a, **b** NEU1 mRNA and protein levels were determined by qRT-PCR (a) and western blots (b) in HK-2 cells stimulated with TGFβ (10 ng/ml) for 24 h. n = 4 samples per group in **a**, n = 3 samples per group in **b**. Data were presented as mean ± SD. Unpaired two-tailed t-test. **c**, **g** *NEU1* mRNA levels determined by RT-qPCR in HK-2 cells. n = 3 samples per group. Data were presented as mean ± SD. One-way ANOVA followed by Tukey's multiple comparisons test. siNEU1-1, siNEU1-2, and siNEU1-3 were 3 different siRNA sequences. HA-NEU1 was the full-length plasmid of NEU1 with HA tag. **d**, **h** NEU1 protein levels determined by western blots. **e**, **f**, **i**, **j** mRNA levels of the indicated genes in HK-2 cells determined by qRT-PCR (**e**, **i**). Indicated protein expression detected by western blots (**f**, **j**). Data were expressed as mean ± SD. n = 4 samples per group in **e**, **i**, n = 3 samples per group in **f**, **j**. Two-way ANOVA followed by Sidak's multiple comparisons test. **k** NEU1 protein in HK-2 cells transfected with NEU1 plasmid detected with native-and SDS-polyacrylamide gel electrophoresis (Native-PAGE) and SDS polyacrylamide gel electrophoresis (SDS-PAGE). Two independent experiments were performed. **c-j** The full-length plasmid and siRNA of NEU1 were transfected into HK-2 cells and treated with or without TGFβ (10 ng/ml) for 24 h. All tests were two-tailed.

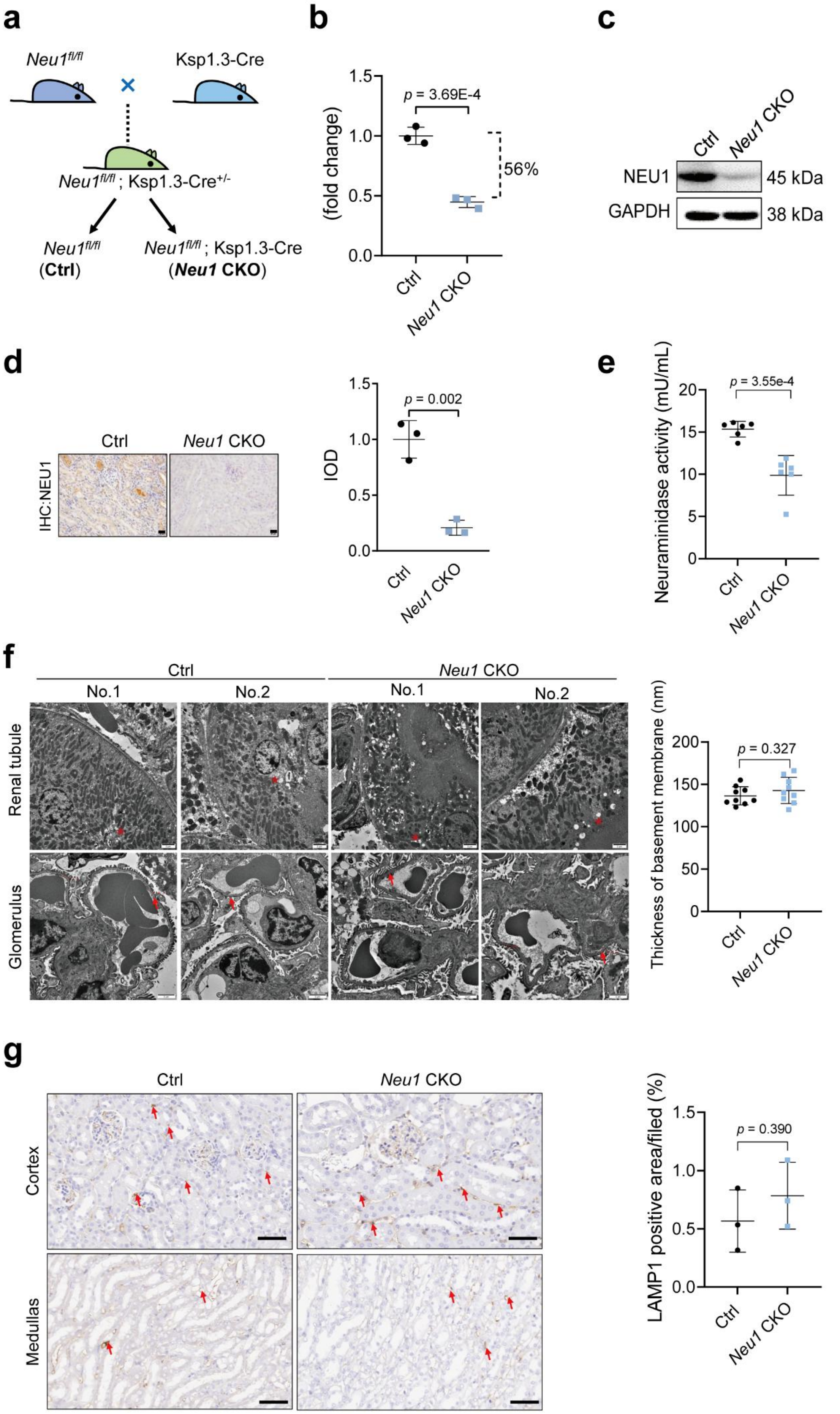
a
Neu1fl/fl
Ksp1.3-Cre
Neu1fl/fl ; Ksp1.3-Cre+/-
Neu1fl/fl (Ctrl)
Neu1fl/fl ; Ksp1.3-Cre (Neu1 CKO)
b
(fold change)
p = 3.69E-4
56%
Ctrl
Neu1 CKO
c
Ctrl
Neu1 CKO
NEU1
45 kDa
GAPDH
38 kDa
d
Ctrl
Neu1 CKO
IHC:NEU1
IOD
p = 0.002
e
Neuraminidase activity (mU/mL)
p = 3.55e-4
f
Ctrl
Neu1 CKO
No.1
No.2
Renal tubule
Glomerulus
Thickness of basement membrane (nm)
p = 0.327
g
Ctrl
Neu1 CKO
Cortex
Medullas
LAMP1 positive area/filed (%)
p = 0.390

Supplementary Fig. 3 Generation of the TECs-specific *Neu1* conditional knockout (*Neu1* CKO) mice.

a Schematic of the strategy to generate *Neu1* CKO mice. **b** Relative mRNA levels of *Neu1* in cortices of kidneys from control (Ctrl) and *Neu1* CKO mice. n = 3 mice per group. **c** Western blots of NEU1 in the kidneys of Ctrl and *Neu1* CKO mice. **d** Images of immunohistochemistry (up) and quantitative results (down). The NEU1 was determined with IOD by Image-Pro Plus 6.0. n = 3 samples per group. Scale bar, 20 μm. IOD: integrated optical density. **e** Neuraminidase enzyme activity in kidneys was measured by a fluorometric assay with substrate 2′-(4-methylumbelliferyl)-α-d-N-acetylneuraminic acid (4-MU-NANA). n = 6 samples per group. **f** Left, representative transmission electron microscopy (TEM) images of kidney tubules and glomerulus from *Neu1*CKO and Ctrl mice. n = 2 mice per group. Scale bar, 2 μm. Right, the thickness of the glomerular basement membrane was calculated from nine glomeruli in two ctrl mice and two *Neu1* CKO mice. The asterisks indicate vacuoles. The arrows indicate glomerular basement membrane. **g** Immunohistochemistry staining of LAMP1 in kidney sections from Ctrl and *Neu1* CKO mice. n = 3 mice per group. All data were presented as mean ± SD. Unpaired two-tailed t-test. All tests were two-tailed.

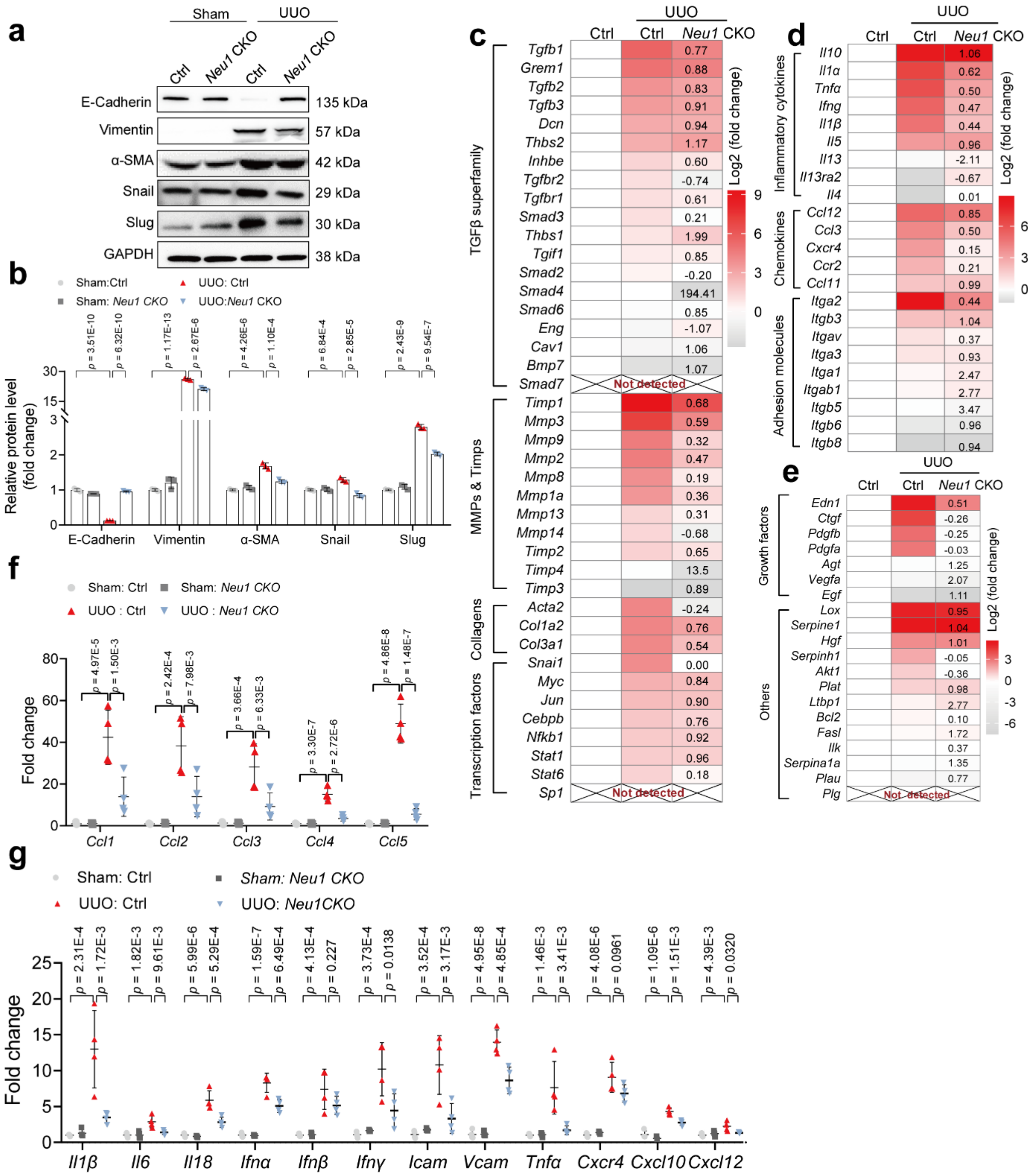
a
Sham
UUO
Ctrl
Neu1 CKO
E-Cadherin
Vimentin
α-SMA
Snail
Slug
GAPDH
135 kDa
57 kDa
42 kDa
29 kDa
30 kDa
38 kDa
b
Sham:Ctrl
Sham: Neu1 CKO
UUO: Ctrl
UUO:Neu1 CKO
Relative protein level (fold change)
E-Cadherin
Vimentin
α-SMA
Snail
Slug
c
UUO
Ctrl
Neu1 CKO
TGFβ superfamily
Tgfb1
Grem1
Tgfb2
Tgfb3
Dcn
Thbs2
Inhbe
Tgfbr2
Tgfbr1
Smad3
Thbs1
Tgif1
Smad2
Smad4
Smad6
Eng
Cav1
Bmp7
Smad7
Not detected
MMPs & Timps
Timp1
Mmp3
Mmp9
Mmp2
Mmp8
Mmp1a
Mmp13
Mmp14
Timp2
Timp4
Timp3
Collagens
Acta2
Col1a2
Col3a1
Transcription factors
Snai1
Myc
Jun
Cebpb
Nfkb1
Stat1
Stat6
Sp1
Log2 (fold change)
d
Inflammatory cytokines
Il10
Il1α
Tnfα
Ifng
Il1β
Il5
Il13
Il13ra2
Il4
Chemokines
Ccl12
Ccl3
Cxcr4
Ccr2
Ccl11
Adhesion molecules
Itga2
Itgb3
Itgav
Itga3
Itga1
Itgab1
Itgb5
Itgb6
Itgb8
e
Growth factors
Edn1
Ctgf
Pdgfb
Pdgfa
Agt
Vegfa
Egf
Others
Lox
Serpine1
Hgf
Serpinh1
Akt1
Plat
Ltbp1
Bcl2
Fasl
Ilk
Serpina1a
Plau
Plg
f
Sham: Ctrl
Sham: Neu1 CKO
UUO : Ctrl
UUO : Neu1 CKO
Fold change
Ccl1
Ccl2
Ccl3
Ccl4
Ccl5
g
Sham: Ctrl
Sham: Neu1 CKO
UUO: Ctrl
UUO: Neu1CKO
Fold change
Il1β
Il6
Il18
Ifnα
Ifnβ
Ifnγ
Icam
Vcam
Tnfα
Cxcr4
Cxcl10
Cxcl12

Supplementary Fig. 4 NEU1 deficiency inhibits UUO-induced EMT and inflammation response.

a, b Western blots (**a**) and quantitative results (**b**) of E-Cadherin, Vimentin, α-SMA, Snail, and Slug in kidneys of control (Ctrl) and *Neu1* CKO mice 10 days after UUO. Relative protein levels were shown after normalization to GAPDH. n = 3 samples per group. Data were presented as mean ± SD from three independent experiments. Two-way ANOVA followed by Tukey's multiple comparisons test. **c-e** Heat map showing the log (fold-changes) in fibrosis genome in TECs-specific *Neu1*CKO mice determined by PCR array analysis. Data in heat map represent the fold change relative to the UUO group. **f**, **g** the mRNA levels of chemokines (*Ccl1, Ccl2, Ccl3, Ccl4,* and *Ccl5*) (**f**) and inflammatory cytokines-associate genes (**g**) in kidney samples, all normalized to *Gapdh*. Data were presented as the mean ± SD. n = 4 samples per group. Two-way ANOVA followed by Tukey's multiple comparisons test. All tests were two-tailed.

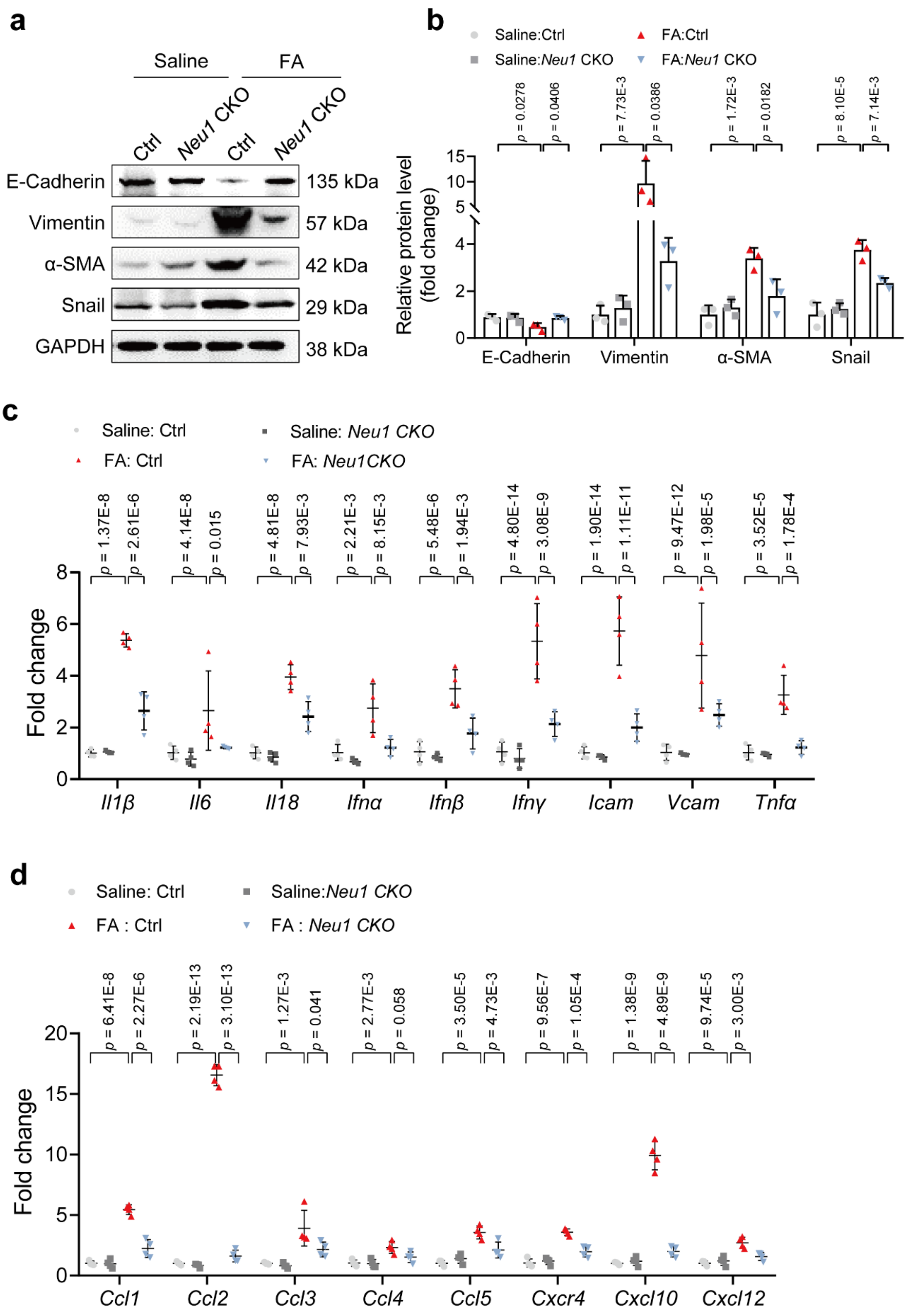
a
Saline
FA
Ctrl
Neu1 CKO
Ctrl
Neu1 CKO
E-Cadherin
Vimentin
α-SMA
Snail
GAPDH
135 kDa
57 kDa
42 kDa
29 kDa
38 kDa
b
Saline:Ctrl
FA:Ctrl
Saline:Neu1 CKO
FA:Neu1 CKO
p = 0.0278
p = 0.0406
p = 7.73E-3
p = 0.0386
p = 1.72E-3
p = 0.0182
p = 8.10E-5
p = 7.14E-3
Relative protein level (fold change)
E-Cadherin
Vimentin
α-SMA
Snail
c
Saline: Ctrl
Saline: Neu1 CKO
FA: Ctrl
FA: Neu1CKO
p = 1.37E-8
p = 2.61E-6
p = 4.14E-8
p = 0.015
p = 4.81E-8
p = 7.93E-3
p = 2.21E-3
p = 8.15E-3
p = 5.48E-6
p = 1.94E-3
p = 4.80E-14
p = 3.08E-9
p = 1.90E-14
p = 1.11E-11
p = 9.47E-12
p = 1.98E-5
p = 3.52E-5
p = 1.78E-4
Fold change
Il1β
Il6
Il18
Ifnα
Ifnβ
Ifnγ
Icam
Vcam
Tnfα
d
Saline: Ctrl
Saline:Neu1 CKO
FA : Ctrl
FA : Neu1 CKO
p = 6.41E-8
p = 2.27E-6
p = 2.19E-13
p = 3.10E-13
p = 1.27E-3
p = 0.041
p = 2.77E-3
p = 0.058
p = 3.50E-5
p = 4.73E-3
p = 9.56E-7
p = 1.05E-4
p = 1.38E-9
p = 4.89E-9
p = 9.74E-5
p = 3.00E-3
Fold change
Ccl1
Ccl2
Ccl3
Ccl4
Ccl5
Cxcr4
Cxcl10
Cxcl12

Supplementary Fig. 5 NEU1 deficiency inhibits folic acid-induced EMT and inflammation response.

a, **b** Western blots (**a**) and quantitative results (**b**) of E-Cadherin, Vimentin, α-SMA, Snail, and Slug in kidneys of control (Ctrl) and *Neu1* CKO mice 28 days after folic acid (250 mg/kg) injection. Relative protein levels were shown after normalization to GAPDH. n = 4 samples per group. Data were presented as mean ± SD from three independent experiments. Two-way ANOVA followed by Tukey's multiple comparisons test. **c**, **d** the mRNA levels of inflammatory cytokines associate genes (**c**) and chemokines (**d**) in kidney samples, all normalized to *Gapdh*. Data were presented as the mean ± SD. n = 4 samples per group. Two-way ANOVA followed by Tukey's multiple comparisons test. All tests were two-tailed.

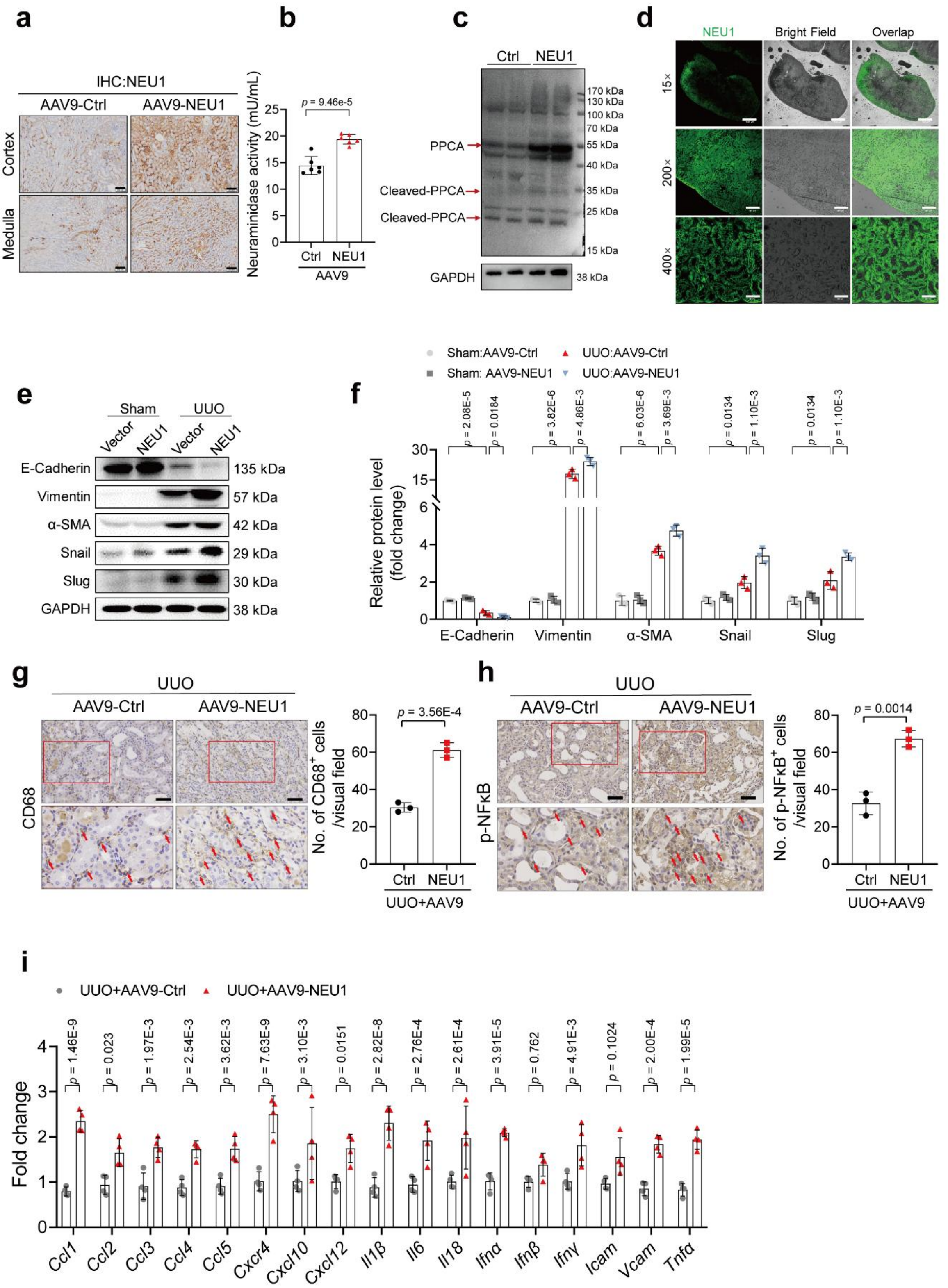
a
IHC:NEU1
AAV9-Ctrl
AAV9-NEU1
Cortex
Medulla
b
p = 9.46e-5
Neuraminidase activity (mU/mL)
Ctrl
NEU1
AAV9
c
Ctrl
NEU1
PPCA
Cleaved-PPCA
Cleaved-PPCA
170 kDa
130 kDa
100 kDa
70 kDa
55 kDa
40 kDa
35 kDa
25 kDa
15 kDa
GAPDH
38 kDa
d
NEU1
Bright Field
Overlap
15×
200×
400×
e
Sham
UUO
Vector
NEU1
Vector
NEU1
E-Cadherin
135 kDa
Vimentin
57 kDa
α-SMA
42 kDa
Snail
29 kDa
Slug
30 kDa
GAPDH
38 kDa
f
Sham:AAV9-Ctrl
UUO:AAV9-Ctrl
Sham: AAV9-NEU1
UUO:AAV9-NEU1
p = 2.08E-5
p = 0.0184
p = 3.82E-6
p = 4.86E-3
p = 6.03E-6
p = 3.69E-3
p = 0.0134
p = 1.10E-3
p = 0.0134
p = 1.10E-3
Relative protein level (fold change)
E-Cadherin
Vimentin
α-SMA
Snail
Slug
g
UUO
AAV9-Ctrl
AAV9-NEU1
CD68
p = 3.56E-4
No. of CD68+ cells /visual field
Ctrl
NEU1
UUO+AAV9
h
UUO
AAV9-Ctrl
AAV9-NEU1
p-NFκB
p = 0.0014
No. of p-NFκB+ cells /visual field
Ctrl
NEU1
UUO+AAV9
i
UUO+AAV9-Ctrl
UUO+AAV9-NEU1
p = 1.46E-9
p = 0.023
p = 1.97E-3
p = 2.54E-3
p = 3.62E-3
p = 7.63E-9
p = 3.10E-3
p = 0.0151
p = 2.82E-8
p = 2.76E-4
p = 2.61E-4
p = 3.91E-5
p = 0.762
p = 4.91E-3
p = 0.1024
p = 2.00E-4
p = 1.99E-5
Fold change
Ccl1
Ccl2
Ccl3
Ccl4
Ccl5
Cxcr4
Cxcl10
Cxcl12
Il1β
Il6
Il18
Ifnα
Ifnβ
Ifnγ
Icam
Vcam
Tnfα

Supplementary Fig. 6 NEU1 overexpression augments UUO-induced renal fibrosis by Adeno-associated virus 9 (AAV9).

a Images of immunolabeling for NEU1 in the indicated experimental groups. Scale bar, 100 μm. **b** Neuraminidase activity in kidneys was measured by a fluorometric assay with substrate 2′-(4-methylumbelliferyl)-α-d-N-acetylneuraminic acid (4-MU-NANA). n = 6 samples per group. Data were presented as mean ± SD. Unpaired two-tailed t-test. **c** Representative western blots of PPCA protein in the kidney of AAV9-Ctrl and AAV9-NEU1 mice. Two independent experiments were performed. **d**, Immunofluorescence images of NEU1 staining in kidney sections from AAV9-NEU1 mice. Scale bar, 1500 μm, 200 μm and 50 μm. Three independent experiments were performed. **e**, **f** Representative western blots (**e**) and quantitative results (**f**) of E-Cadherin, Vimentin, α-SMA, Snail, and Slug in the kidney of AAV9-Ctrl and AAV9-NEU1 mice. Relative protein levels were shown after normalization to GAPDH. n = 3 mice per group. Data were presented as mean ± SD from three independent experiments. Two-way ANOVA followed by Tukey's multiple comparisons test. **g**, **h** Immunohistochemistry staining of CD68 or p-NFκB in kidney sections (g, left; h, left) and quantitative results (g, right; h, right) from AAV9-Ctrl and AAV9-NEU1 mice 10 days after UUO. n = 3 mice per group. Data are presented as mean ± SD. Unpaired two-tailed t-test. **i** the mRNA levels of chemokines (*Ccl1*, *Ccl2*, *Ccl3*, *Ccl4*, *Ccl5*, *Cxcr10*, and *Cxcr12*), inflammatory cytokines (*Il1β*, *Il6*, and *Il18*), interferons (*Ifnα*, *Ifnβ*, and *Ifnγ*), adhesion molecules (*Icam* and *Vcam*), and tumor necrosis factor (*Tnfα*) in kidney samples, all normalized to *Gapdh*. Data were presented as the mean ± SD. n = 4 samples per group. Unpaired two-tailed t-test.

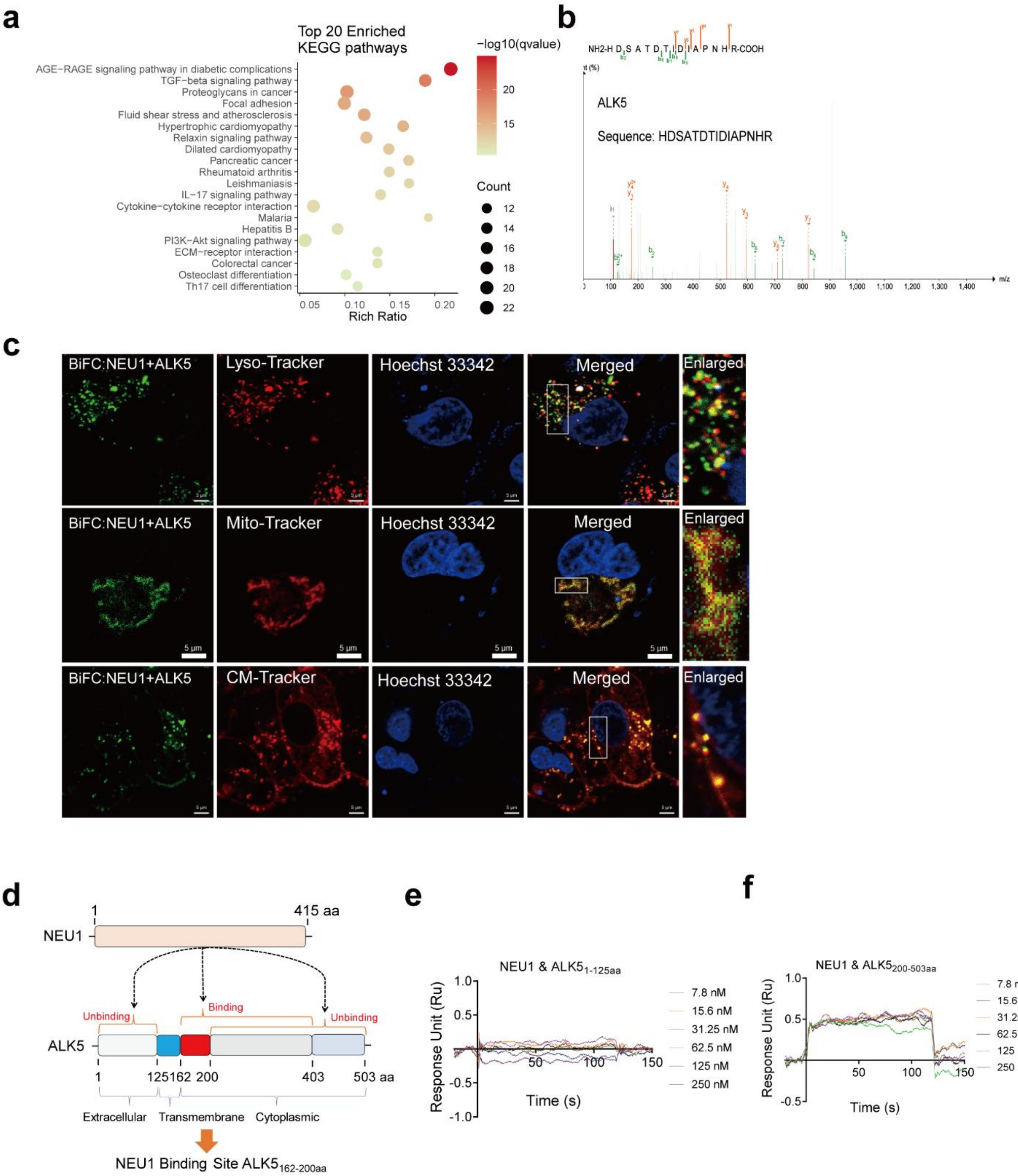
a
Top 20 Enriched KEGG pathways
AGE−RAGE signaling pathway in diabetic complications
TGF−beta signaling pathway
Proteoglycans in cancer
Focal adhesion
Fluid shear stress and atherosclerosis
Hypertrophic cardiomyopathy
Relaxin signaling pathway
Dilated cardiomyopathy
Pancreatic cancer
Rheumatoid arthritis
Leishmaniasis
IL−17 signaling pathway
Cytokine−cytokine receptor interaction
Malaria
Hepatitis B
PI3K−Akt signaling pathway
ECM−receptor interaction
Colorectal cancer
Osteoclast differentiation
Th17 cell differentiation
Rich Ratio
−log10(qvalue)
Count
b
NH2-H D S A T D T I D I A P N H R-COOH
ALK5
Sequence: HDSATDTIDIAPNHR
m/z
c
BiFC:NEU1+ALK5
Lyso-Tracker
Hoechst 33342
Merged
Enlarged
Mito-Tracker
CM-Tracker
5 μm
d
NEU1
1
415 aa
Unbinding
Binding
Unbinding
ALK5
1
125
162
200
403
503 aa
Extracellular
Transmembrane
Cytoplasmic
NEU1 Binding Site ALK5162-200aa
e
NEU1 & ALK51-125aa
Response Unit (Ru)
Time (s)
7.8 nM
15.6 nM
31.25 nM
62.5 nM
125 nM
250 nM
f
NEU1 & ALK5200-503aa
Response Unit (Ru)
Time (s)
7.8 nM
15.6 nM
31.25 nM
62.5 nM
125 nM
250 nM

Supplementary Fig. 7 NEU1 interacts with ALK5.

a Kyoto Encyclopedia of Genes and Genomes (KEGG) pathway enrichment analysis of significantly changed genes of PCR array. The top 20 enriched KEGG pathways were showed. **b** Liquid chromatography-tandem mass spectrometry (LC-MS/MS) scans of proteins that bind to NEU1 in HK-2 cells were performed. Representative LC-MS/MS spectra showed the peptide fragmentation of ALK5. **c** Representative fluorescence images of HK-2 cells co-expression of NEU1-VC155 and ALK5-VN173 plasmid with TGFβ stimulation. Lyso-Tracker, Mito-Tracker, and CM-Tracker were used to label lysosome, mitochondrion, and cell plasma membrane respectively. Scale bar, 5 μm. Three independent experiments were repeated with similar results. **d** Schematic diagram of NEU1 binding with ALK5. **e** The interaction between NEU1 and $ALK5_{1\text{-}125aa}$ was tested by SPR. **f** The interaction between NEU1 and $ALK5_{200\text{-}503aa}$ was tested by SPR.

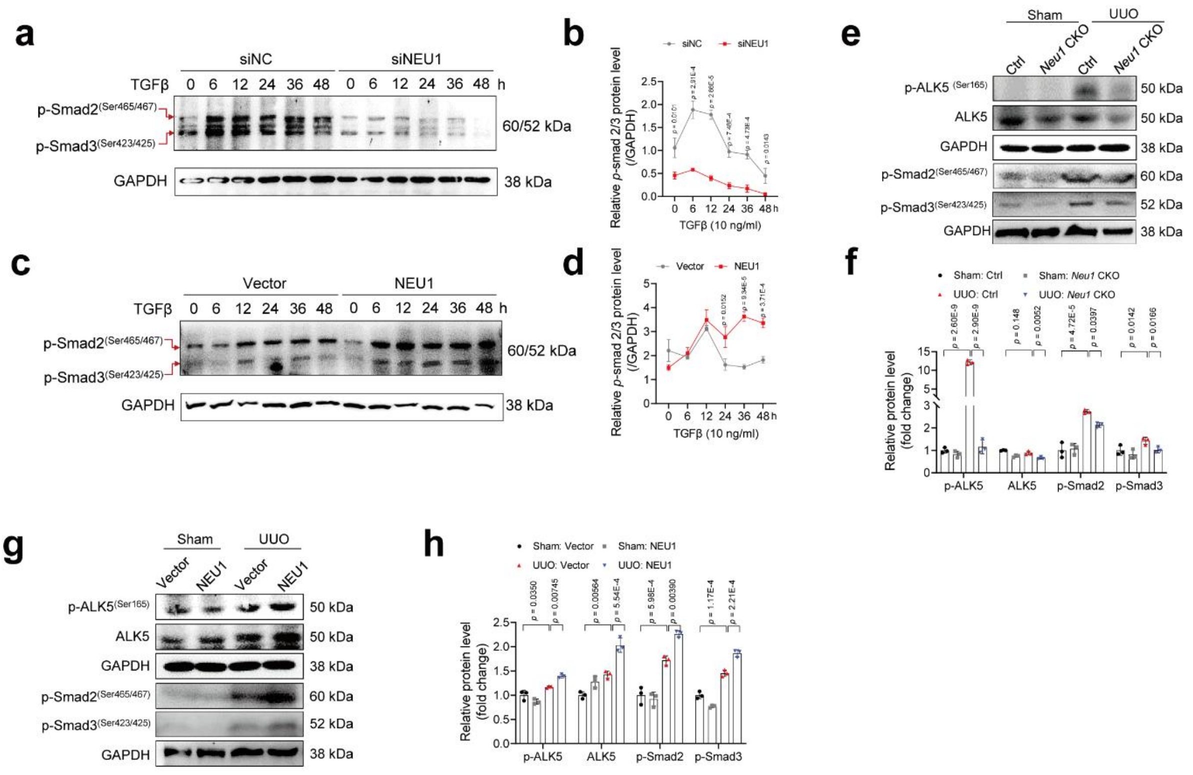

Supplementary Fig. 8 NEU1 enhances ALK5-SMAD2/3 signaling pathway in response to TGFβ stimulation in HK-2 cells.

a-**d** HK-2 cells transfected with siNEU1 (**a**, **b**) or NEU1 plasmid (**c**, **d**) for 24 h. Then the cells were stimulated with TGFβ (10 ng/ml) for the indicated periods of time (0, 6, 12, 24, 36, 48 h). Lysates were harvested from the cells and analyzed by western blots (**a**, **c**) Quantitation of the protein levels were shown in **b**, **d**. Data were presented as the mean ± SD. *n* = 3 samples per group. Unpaired two-tailed *t*-test. **e-h,** Western blots and quantitation of p-ALK5 (Ser165), ALK5, p-SMAD2(Ser465/467), and p-SMAD3 (Ser423/425) protein in kidney from *Neu1* CKO (**e, f**) or NEU1 overexpressed (**g, h**) mice subjected to UUO for 10 days. Data were presented as the mean ± SD. *n* = 3 samples per group. One-way ANOVA followed by Tukey's multiple comparisons test. All tests were two-tailed.

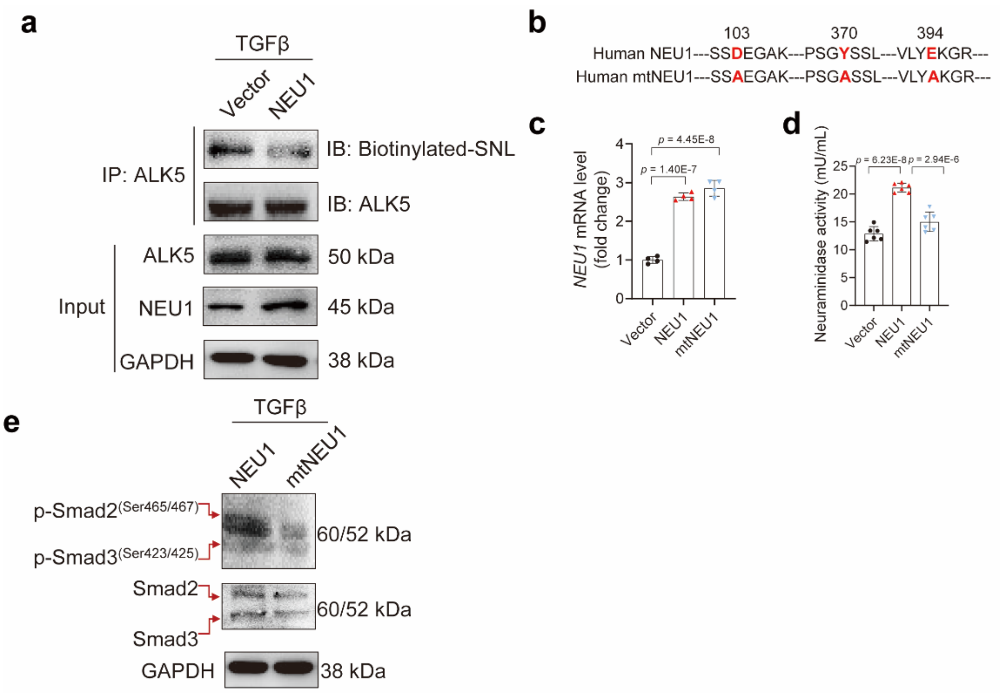

Supplementary Fig. 9 NEU1 promotes the desialylation of ALK5 and inactivation of NEU1 inhibits Smad2/3 phosphorylation in HK-2 cells.

a The sialylation level of ALK5 was detected by measuring the alpha 2-6-linked sialic acid bond (biotinylated sambucus nigra lectin, SNL) expression. Three independent experiments were repeated with similar results. **b** Human NEU1 amino acid mutation site (mtNEU1: D103A, Y370A, E394A). **c** Relative mRNA levels of *Neu1* in HK-2 cells transfected with NEU1 and mtNEU1 plasmid. *n* = 4 samples per group. Data were presented as mean ± SD. One-way ANOVA followed by Tukey's multiple comparisons test. **d** Neuraminidase activity in HK-2 cells was measured by a fluorometric assay with substrate 2′-(4-methylumbelliferyl)-α-d-N-acetylneuraminic acid (4-MU-NANA). *n* = 6 samples per group. Data were presented as mean ± SD. One-way ANOVA followed by Tukey's multiple comparisons test. **e** Representative western blots of p-Smad2 (Ser465/467), p-Smad3 (Ser423/425), and Smad2/3, in HK-2 cells transfected with NEU1 and mtNEU1 plasmid. Three independent experiments were repeated with similar results. All tests were two-tailed.

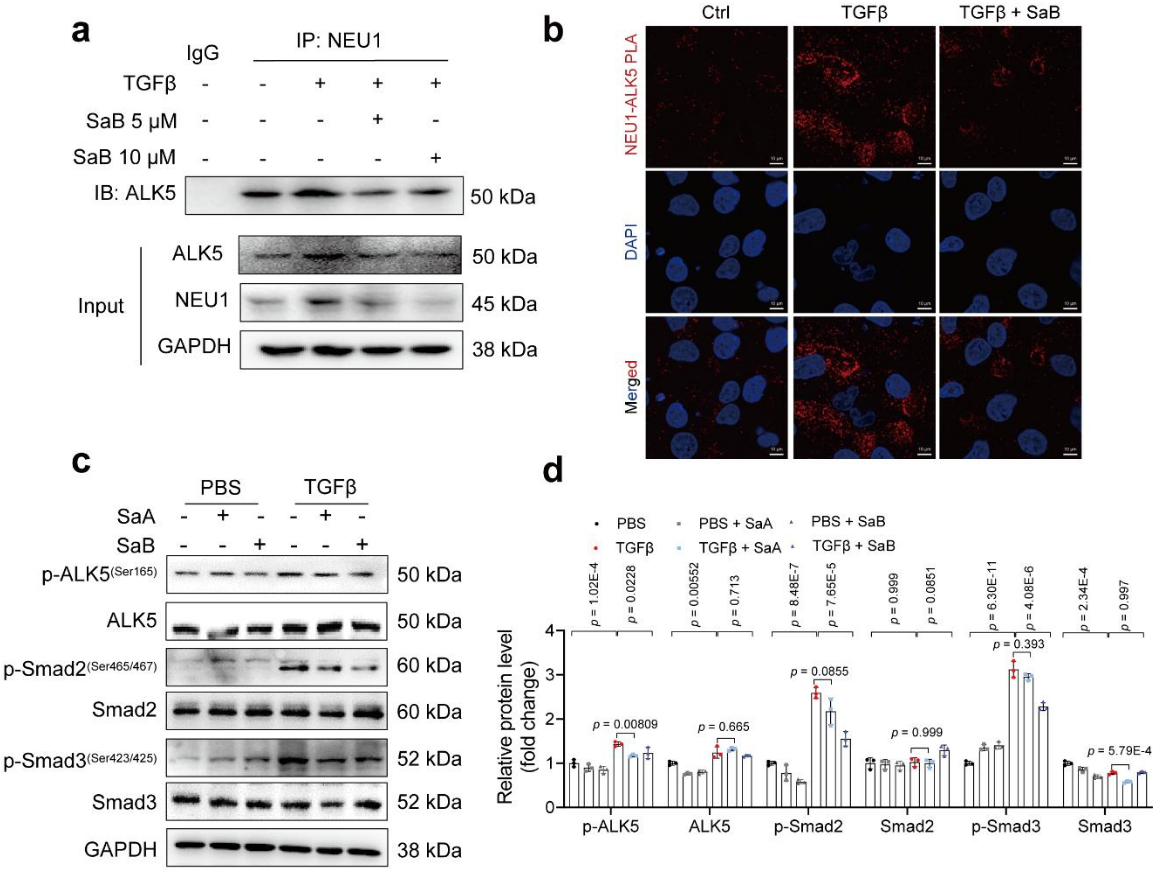

Supplementary Fig. 10 Salvianolic acid B inhibits NEU1 binding to ALK5 and suppresses ALK5-Smad2/3 signal pathway.

a Co-immunoprecipitation of NEU1 and ALK5 in HK-2 cells treated with salvianolic acid B (SaB, 5 µM, 10 µM) in response to TGFβ for 24h. Two independent experiments were repeated with similar results. **b** Interaction between NEU1 and ALK5 (NEU1-ALK5, red) was analyzed by PLA in HK-2 cells treated with SaB (10 µM) in response to TGFβ for 24h. Scale bars, 10 µm. n = 3 samples per group. **c**, **d** Representative western blots (**c**) and quantitative results (**d**) of p-ALK5(Ser165), p-SMAD2(Ser465/467), SMAD2/3, and p-SMAD3 (Ser423/425) in HK-2 cells treated with salvianolic acid A (SaA, 10 µM) and salvianolic acid B (10 µM). Relative protein levels were shown after normalization to GAPDH. n = 3 mice per group. Data were presented as mean ± SD. One-way ANOVA followed by Tukey's multiple comparisons test.

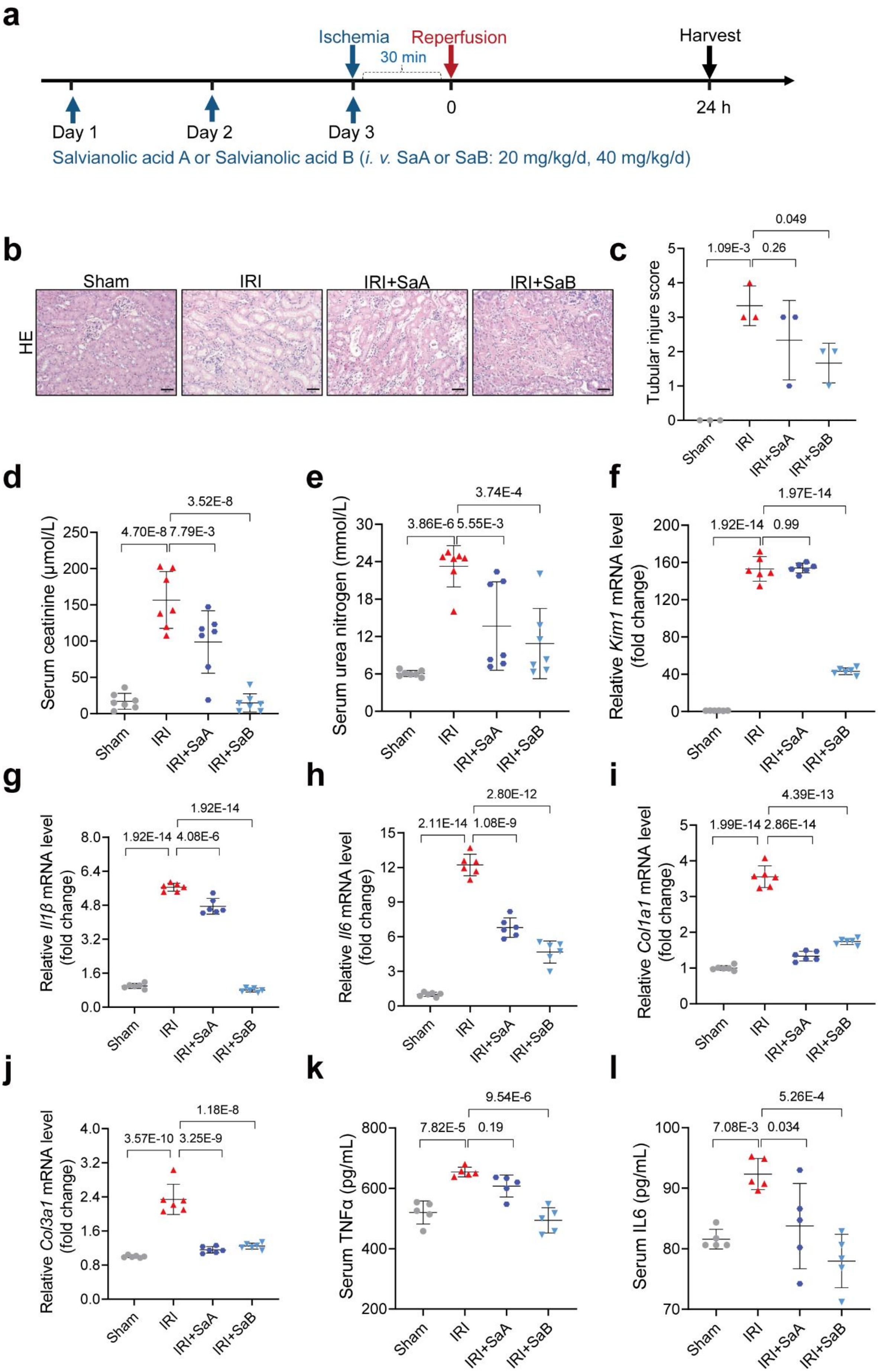
a
Ischemia
Reperfusion
30 min
Harvest
0
24 h
Day 1
Day 2
Day 3
Salvianolic acid A or Salvianolic acid B (i. v. SaA or SaB: 20 mg/kg/d, 40 mg/kg/d)
b
Sham
IRI
IRI+SaA
IRI+SaB
HE
c
Tubular injure score
1.09E-3
0.26
0.049
d
Serum ceatinine (μmol/L)
4.70E-8
7.79E-3
3.52E-8
e
Serum urea nitrogen (mmol/L)
3.86E-6
5.55E-3
3.74E-4
f
Relative Kim1 mRNA level (fold change)
1.92E-14
0.99
1.97E-14
g
Relative Il1β mRNA level (fold change)
1.92E-14
4.08E-6
1.92E-14
h
Relative Il6 mRNA level (fold change)
2.11E-14
1.08E-9
2.80E-12
i
Relative Col1a1 mRNA level (fold change)
1.99E-14
2.86E-14
4.39E-13
j
Relative Col3a1 mRNA level (fold change)
3.57E-10
3.25E-9
1.18E-8
k
Serum TNFα (pg/mL)
7.82E-5
0.19
9.54E-6
l
Serum IL6 (pg/mL)
7.08E-3
0.034
5.26E-4

Supplementary Fig. 11 Salvianolic acid B alleviates IR-induced renal injure.

a Scheme of the experimental approach. **b** Histological analysis of kidney slices by hematoxylin and eosin (HE). *n* = 3 mice per group. Scale bar, 50 μm. **c** Statistical analysis of tubular injury score. *n* = 3 mice per group. **d**, **e** Blood urea nitrogen (**d**) and creatinine (**e**) in serum measured by ELISA. *n* = 7 mice per group. **f**-**j** the mRNA levels of *Kim1* (**f**), inflammatory cytokines (**g**, **h**) and extracellular matrix (**i**, **j**) in kidney samples, all normalized to *Gapdh*. *n* = 6 samples per group. **k**, **l** TNFα (**k**) and IL6 (**l**) in serum measured by ELISA. *n* = 5 samples per group. All statistic data were presented as mean ± SD, one-way ANOVA followed by Tukey's multiple comparisons test. All tests were two-tailed.

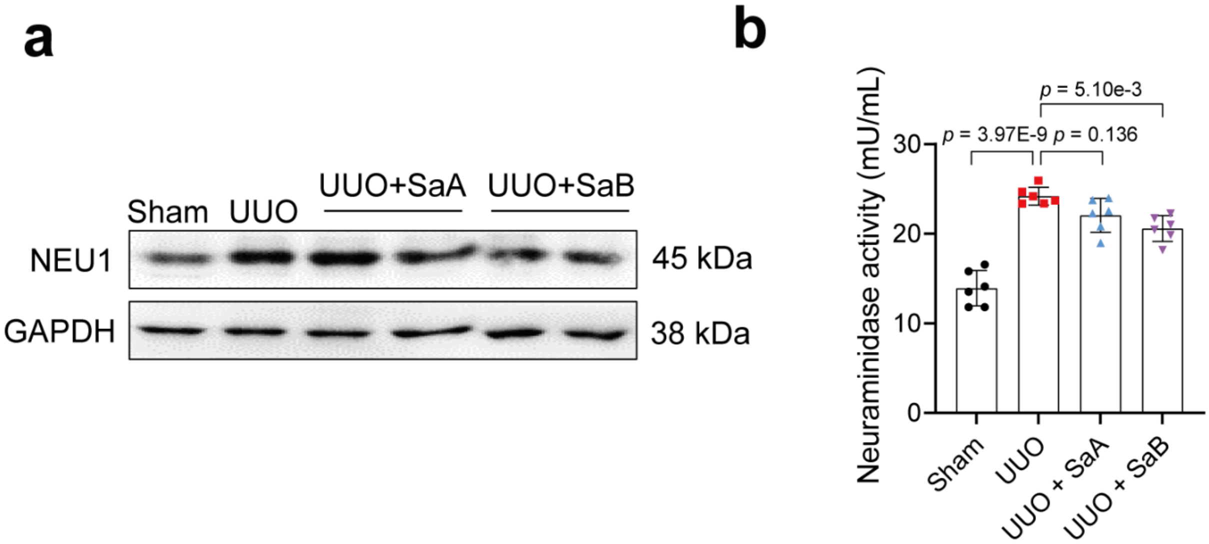

Supplementary Fig. 12 Salvianolic acid B inhibits NEU1 expression.

a Representative western blot of NEU1 protein in kidney from salvianolic acid A or salvianolic acid B treated mice subjected to UUO for 10 days. **b** Neuraminidase activity in kidneys was measured by a fluorometric assay with substrate 2′-(4-methylumbelliferyl)-α-d-N-acetylneuraminic acid (4-MU-NANA). *n* = 6 samples per group. Data were presented as mean ± SD. One-way ANOVA followed by Tukey's multiple comparisons test.

Supplementary Table 1. Clinical information of the subjects

**Characteristics**	**Renal Fibrosis (*n* = 8)**	**Non-Renal Fibrosis (*n* = 8)**	***p* value**
Age (years)	39.00 ± 8.18	58.75 ± 8.41	0.00055*
Male (n, %)	5 (62.50%)	5 (62.50%)	1.0000[#]
SCr (μmol/L)	307.3 ± 156.1	68.81 ± 15.58	0.0013*
BUN (mmol/L)	15.25 ± 6.59	4.920 ± 1.59	0.0012*
GFR (ml/min)	23.50 ± 9.12	94.63 ± 13.60	1.63e-8*
Comorbidities/Medical history			
Hypertension	7 (87.5%)	4 (50.0%)	
Diabetes mellitus	0	1 (12.5%)	
COPD or asthma	0	0	
Coronary disease	0	2 (25.0%)	
Morbid obesity	0	0	
Neurologic	0	0	
Neoplasms (extra-renal)	0	1 (12.5%)	

#Fisher's two-tailed test. *Unpaired two-tailed *t*-test. SCr: serum creatinine; BUN: Blood urea nitrogen. GFR: Glomerular filtration rate; COPD: chronic obstructive pulmonary disease.

Supplementary Table 2. List of siRNA

Gene names	Sequence
siNEU1-1	sense: CCAAGGCUGAGAACGACUUTT
	antisense: AAGUCGUUCUCAGCCUUGGTT
siNEU1-2	sense: GGAAUCUCUCCCUGGAUAUTT
	antisense: AUAUCCAGGGAGAGAUUCCTT
siNEU1-3	sense: GGCUAUUCAUCCCUGGCAATT
	antisense: UUGCCAGGGAUGAAUAGCCTT
siALK5-1	sense: CCAACUACUGUAAAGUCAUTT
	antisense: AUGACUUUACAGUAGUUGGTT
siALK5-2	sense: CCAUUGAUAUUGCUCCAAATT
	antisense: UUUGGAGCAAUAUCAAUGGTT
siALK5-3	sense: GCUUACAGCAUUGCGGAUUTT
	antisense: AAUCCGCAAUGCUGUAAGCTT

Supplementary Table 3. List of primers used for qRT-PCR

Gene names	Primer	Sequence
Human *ALK5*	Forward	5'-GTGACAGATGGGCTCTGCTT-3'
Human *ALK5*	Reversed	5'-AGGGCCAGTAGTTGGAAGTT-3'
Human *CDH1*	Forward	5'-AGTCACTGACACCAACGATAAT-3'
Human *CDH1*	Reversed	5'-ATCGTTGTTCACTGGATTTGTG-3'
Human *FN1*	Forward	5'- CCGGGACTCAATCCAAATGC-3'
Human *FN1*	Reversed	5'- TCCGTAGGTTGGTTCAAGCC-3'
Human *KIM1*	Forward	5'-CCCACGTCACCTATCGGAAG-3'
Human *KIM1*	Reversed	5'-GTGCTCAACACGGCAACAAT-3'
Human *NEU1*	Forward	5'-CGAATTGTCCTCCGCAGCTA-3'
Human *NEU1*	Reversed	5'-TCCGCCATGAGGTACCATTG-3'
Human *SNAI1*	Forward	5'-CCTCGCTGCCAATGCTCATCTG-3'
Human *SNAI1*	Reversed	5'-GCTCTGCCACCCTGGGACTC-3'
Human *SNAI2*	Forward	5'-GGCTCATCTGCAGACCCATT-3'
Human *SNAI2*	Reversed	5'-TGCTACACAGCAGCCAGATT-3'
Human *VIM*	Forward	5'-GGACCAGCTAACCAACGACA-3'
Human *VIM*	Reversed	5'-AAGGTCAAGACGTGCCAGAG-3'
Human *GAPDH*	Forward	5'-GTCAAGGCTGAGAACGGGAA-3'
Human *GAPDH*	Reversed	5'-AAATGAGCCCCAGCCTTCTC-3'
Mouse *Acta2*	Forward	5'-CTTCGTGACTACTGCCGAGC-3'
Mouse *Acta2*	Reversed	5'-TGCATCCTGTCAGCAATGCCT-3'
Mouse *Ccl1*	Forward	5'-CTTCCCCTGAAGTTTATCCAGT-3'
Mouse *Ccl1*	Reversed	5'-TCTACCTTTGTTCAGCCTGAAT-3'
Mouse *Ccl2*	Forward	5'-TTTTTGTCACCAAGCTCAAGAG-3'
Mouse *Ccl2*	Reversed	5'-TTCTGATCTCATTTGGTTCCGA-3'
Mouse *Ccl3*	Forward	5'-TTGCTGTTCTTCTCTGTACCAT-3'
Mouse *Ccl3*	Reversed	5'-AATAGTCAACGATGAATTGGCG-3'
Mouse *Ccl4*	Forward	5'-ACTTCCTGCTGTTTCTCTTACA-3'
Mouse *Ccl4*	Reversed	5'-CCAAGTCACTCATGTACTCAGT-3'
Mouse *Ccl5*	Forward	5'-GTATTTCTACACCAGCAGCAAG-3'
Mouse *Ccl5*	Reversed	5'-TCTTGAACCCACTTCTTCTCTG-3'
Mouse *Cdh1*	Forward	5'-CTGTGAAGGGACGGTCAACA-3'
Mouse *Cdh1*	Reversed	5'-ATCAGAATCAGCAGGGCGAG-3'
Mouse *Co1a1*	Forward	5'-CTGACGCATGGCCAAGAAGA-3'
Mouse *Col1a1*	Reversed	5'-CGTGCCATTGTGGCAGATAC-3'
Mouse *Col3a1*	Forward	5'-TGACTGTCCCACGTAAGCAC-3'
Mouse *Col3a1*	Reversed	5'-GGAGGGCCATAGCTGAACTG-3'
Mouse *Cxcr4*	Forward	5'-CTCATCCTAGCTTTCTTTGCCT-3'
Mouse *Cxcr4*	Reversed	5'-GAAGTCACATCCTTGCTTGATG-3'
Mouse *Cxcl10*	Forward	5'-CAACTGCATCCATATCGATGAC-3'
Mouse *Cxcl10*	Reversed	5'-GATTCCGGATTCAGACATCTCT-3'
Mouse *Cxcl12*	Forward	5'-TCTGAAAATCCTCAACACTCCA-3'
Mouse *Cxcl12*	Reversed	5'-CAGGTACTCTTGGATCCACTTT-3'

Mouse *Fn1*	Forward	5'-GAAGTCGCAAGGAAACAAGC-3'
Mouse *Fn1*	Reversed	5'-GCCACCATAAGTCTGGGTCA-3'
Mouse *Icam*	Forward	5'-CTGAAAGATGAGCTCGAGAGTG-3'
Mouse *Icam*	Reversed	5'-AAACGAATACACGGTGATGGTA-3'
Mouse *Il1β*	Forward	5'-ATCTCGCAGCAGCACATCAA-3'
Mouse *Il1β*	Reversed	5'-ATGGGAACGTCACACACCAG-3'
Mouse *Il6*	Forward	5'-CTCCCAACAGACCTGTCTATAC-3'
Mouse *Il6*	Reversed	5'-CCATTGCACAACTCTTTTCTCA-3'
Mouse *Il18*	Forward	5'-AGACCTGGAATCAGACAACTTT-3'
Mouse *Il18*	Reversed	5'-TCAGTCATATCCTCGAACACAG-3'
Mouse *Ifnα*	Forward	5'-GACCTGCAAGGCTGTCTGAT-3'
Mouse *Ifnα*	Reversed	5'-AGACAGGGCTCTCCAGACTT-3'
Mouse *Ifnβ*	Forward	5'-CTGGGTGGAATGAGACTATTGT-3'
Mouse *Ifnβ*	Reversed	5'-AAGTTCCTGAAGATCTCTGCTC-3'
Mouse *Ifnγ*	Forward	5'-CTTGAAAGACAATCAGGCCATC-3'
Mouse *Ifnγ*	Reversed	5'-CTTGGCAATACTCATGAATGCA-3'
Mouse *Kim1*	Forward	5'-GCTGCTACTGCTCCTTGTGA-3'
Mouse *Kim1*	Reversed	5'-GGAAGGCAACCACGCTTAGA-3'
Mouse *Neu1*	Forward	5'-CGTGGTCCTCTACAGCCTTC-3'
Mouse *Neu1*	Reversed	5'-GCCGTCGTCCTTACTCCAAA-3'
Mouse *Snai1*	Forward	5'-CCGGAAGCCCAACTATAGCG-3'
Mouse *Snai1*	Reversed	5'-TGGGGTACCAGGAGAGAGTC-3'
Mouse *Snai2*	Forward	5'-TACAGCGAACTGGACACACA-3'
Mouse *Snai2*	Reversed	5'-GTAAAGGAGAGTGGAGTGGAGC-3'
Mouse *Tgfβ*	Forward	5'-CCAGATCCTGTCCAAACTAAGG-3'
Mouse *Tgfβ*	Reversed	5'-CTCTTTAGCATAGTAGTCCGCT-3'
Mouse *Tnc*	Forward	5'-GCTTTGTTTGCCCTCACTCC-3'
Mouse *Tnc*	Reversed	5'-GGGTCATGTTTAGCCCACTCT-3'
Mouse *Tnfα*	Forward	5'-ATGTCTCAGCCTCTTCTCATTC-3'
Mouse *Tnfα*	Reversed	5'-GCTTGTCACTCGAATTTTGAGA-3'
Mouse *Vim*	Forward	5'-GAGCTATGTGACCACGTCCA-3'
Mouse *Vim*	Reversed	5'-CCGGGGGATGAGGAATAGAG-3'
Mouse *Vcam*	Forward	5'-CTGAAAGATGAGCTCGAGAGTG-3'
Mouse *Vcam*	Reversed	5'-AAACGAATACACGGTGATGGTA-3'
Mouse *Gapdh*	Forward	5'-CCCTTATTGACCTCAACTACATGGT-3'
Mouse *Gapdh*	Reversed	5'-GAGGGGCCATCCACAGTCTTCTG-3'

Supplementary Table 4. List of compounds for SPR

Number	Compounds	Source	KD (μM)	Chi^2	SE	Purity
1	Panax quinquefolium polysaccharides I	Author's laboratory	48.42	34.7	0.0014	≥90%
2	Panax quinquefolium polysaccharides II	Author's laboratory	89.53	35.1	1.20E-4	≥90%
3	Panax quinquefolium polysaccharides III	Author's laboratory	ND	2.86	7.40E-4	≥90%
4	Panax quinquefolium polysaccharides IV	Author's laboratory	11.81	27.7	0.002	≥90%
5	Panax quinquefolium polysaccharides V	Author's laboratory	14.81	29.4	5.2E-5	≥90%
6	Panax quinquefolium polysaccharides VI	Author's laboratory	23.5	1.37	4.60E-5	≥90%
7	Panax quinquefolium polysaccharides VII	Author's laboratory	6.39	3.06	4.60E-5	≥90%
8	Panax quinquefolium polysaccharides VIII	Author's laboratory	27.96	200	7.00E-5	≥90%
9	Panax quinquefolium polysaccharides IX	Author's laboratory	ND	9.88	7.30E-4	≥90%
10	Glehniae radix polysaccharide I	Author's laboratory	17.14	16.5	8.80E-4	≥90%
11	Glehniae radix polysaccharide II	Author's laboratory	ND	4.32	3.20E-4	≥90%
12	Glehniae radix polysaccharide III	Author's laboratory	ND	12.5	8.30E-4	≥90%
13	Glehniae radix polysaccharide IV	Author's laboratory	0.24	9.43	3.40E-4	≥90%
14	Seaweed polysaccharide I	Author's laboratory	620.5	117	1.20E-4	≥90%
15	Seaweed polysaccharide II	Author's laboratory	214.9	1.40	5.10E-5	≥90%
16	Seaweed polysaccharide III	Author's laboratory	286.9	9.53	4.50E-4	≥90%
17	Asterias polysaccharides I	Author's laboratory	25.99	5.39	1.60E-4	≥90%
18	Asterias polysaccharides II	Author's laboratory	ND	18.0	0.024	≥90%
19	Asterias polysaccharides III	Author's laboratory	ND	8.19	0.0044	≥90%
20	Asterias polysaccharides IV	Author's laboratory	ND	18.8	0.007	≥90%
21	Asterias polysaccharides V	Author's laboratory	ND	538	0.0019	≥90%

22	Ginsenoside Rb1	MUST	10.48	10.3	9.60E-4	≥98%
23	Geniposide	MUST	4.27	5.99	5.90E-4	≥98%
24	Genipin	MUST	4.83	6.16	6.70E-4	≥98%
25	Chlorogenic acid	MUST	4.33	6.64	0.0013	≥98%
26	Berberine	MUST	144.6	17.5	0.0016	≥98%
27	Notoginsenoside R1	MUST	3.42	159	5.80E-4	≥98%
28	Ginsenoside Rg1	MUST	20.15	10.3	0.0059	≥98%
29	Ginsenoside Rd	MUST	14.49	10.2	0.0014	≥98%
30	Ginsenoside Re	MUST	8.87	8.09	9.10E-4	≥98%
31	TanshinoneⅡA	MUST	8.75	7.47	5.60E-4	≥98%
32	Cryptotanshinone	MUST	7.66	9.15	5.30E-4	≥98%
33	Dihydrotanshinone	MUST	9.51	6.46	0.001	≥98%
34	Jatrorrhizine	MUST	25.32	15.0	5.50E-4	≥98%
35	Baicalin	MUST	7.31	7.60	8.40E-4	≥98%
36	Astragaloside A	MUST	101.1	4.10	8.40E-4	≥98%
37	Cynaroside	MUST	6.08	9.99	7.10E-4	≥98%
38	Luteolin	MUST	2.94	21.0	8.30E-4	≥98%
39	Oleanolic acid	MUST	16.69	10.8	5.50E-4	≥98%
40	macranthoidin B	MUST	10.97	8.36	0.0014	≥98%
41	macranthoidin A	MUST	ND	6.61	4.3	≥98%
42	Macranthoside B	MUST	9.67	15.6	0.0037	≥98%
43	Isochlorogenic acid A	MUST	6.93	6.16	0.001	≥98%

44	Salvianic acid A	MUST	4.33	0.0248	0.0032	≥98%
45	Salvianolic acid A	MUST	52.3	1.18	2.5E-5	≥98%
46	Salvianolic acid B	MUST	0.0216	0.0859	2.4E-11	≥98%
47	Rosmarinic acid	MUST	0.141	0.0003	2.7E-11	≥98%
48	Lithospermic acid	MUST	0.345	0.157	1.9E-10	≥98%
49	Fraxetin	MUST	1.09	9.40	0.004	≥98%
50	Carnosic acid	MUST	62.8	2.85	0.0073	≥98%
51	Andrographolide	MUST	2.28	3.08	0.067	≥98%
52	Kaempferol	MUST	16.51	2.78	0.047	≥98%
53	Curcumol	MUST	ND	6.07	1.5E-4	≥98%
54	Alantolactone	MUST	11.16	2.57	9E-4	≥98%
55	Shikonin	MUST	4.72	2.07	0.0014	≥98%
56	Polydatin	MUST	13.76	1.89	0.0018	≥98%
57	Alpinetin	MUST	13.04	1.98	0.0012	≥98%
58	Salicylic acid	MUST	ND	6.93	0.012	≥98%
59	Rhoifolin	MUST	24.83	1.92	8.2E-4	≥98%
60	Tectorigenin	MUST	ND	5.98	0.0029	≥98%
61	stevioside	MUST	ND	10.5	0.0018	≥98%
62	Atractylodin	MUST	ND	8.87	0.32	≥98%
63	Gambogic acid	MUST	95.01	4.28	0.0054	≥98%
64	Sennoside A	MUST	29.37	3.96	0.0019	≥98%
65	Salicin	MUST	11.01	1.60	0.045	≥98%

66	Icariin	MUST	23.69	3.64	0.008	≥98%
67	Juglone	MUST	ND	4.04	0.011	≥98%
68	DiosMetin	MUST	13.72	6.83	0.0024	≥98%
69	Ginkgolide A	MUST	14.26	3.40	0.0027	≥98%
70	Parthenolide	MUST	13.35	1.59	0.0025	≥98%
71	Tubeimiside Ⅰ	MUST	271.7	3.47	0.065	≥98%
72	Vanillic acid	MUST	ND	4.57	0.004	≥98%
73	Atractylenolide I	MUST	ND	1.64	0.026	≥98%
74	Toosendanin	MUST	13.71	1.83	0.0035	≥98%

Kd: Dissociation equilibrium constant; Chi^2: Chi-square two-tailed test. SE: Standard error. MUST: CHENG DU MUST BIO-TECHNOLOGY CO., LTD. ND: Not detected.
