# Supplementary material for: Neuraminidase 1 promotes renal fibrosis development in male mice
Source: Nat Commun. 2023 Mar 27;14:1713. doi: 10.1038/s41467-023-37450-8 (PMC10043283; doi:10.1038/s41467-023-37450-8)
Supplement: Supplementary file 2 — Description of Additional Supplementary Files [file 41467_2023_37450_MOESM2_ESM.pdf]

Description of Additional Supplementary Files

Supplementary Data 1: Proteins interacting with the human NEU1 protein were identified by the immunoprecipitation-mass spectrometry (IP-MS). HK-2 cells were stimulated with TGFβ for 24 h. The cells were lysed and immunoprecipitated with anti-IgG or anti-NEU1 antibody. The immunoprecipitant protein was then subjected to liquid chromatography with tandem mass spectrometry for proteomics analysis. LFQ, label-free quantification.

Supplementary Data 2: Screening of NEU1-bound compounds by SPR

The compounds 1-21 are polysaccharides extracted by Wang Peipei's research group, and the compounds 22-74 are commercial small-molecule compounds. The structure of small molecule compounds was shown. Kd, dissociation constant.
