# Supplementary figures and images for: Neuraminidase 1 promotes renal fibrosis development in male mice
Source: Nat Commun. 2023 Mar 27;14:1713. doi: 10.1038/s41467-023-37450-8 (PMC10043283; doi:10.1038/s41467-023-37450-8)

## Supplementary Data 2 Screening of NEU1-bound compounds by SPR

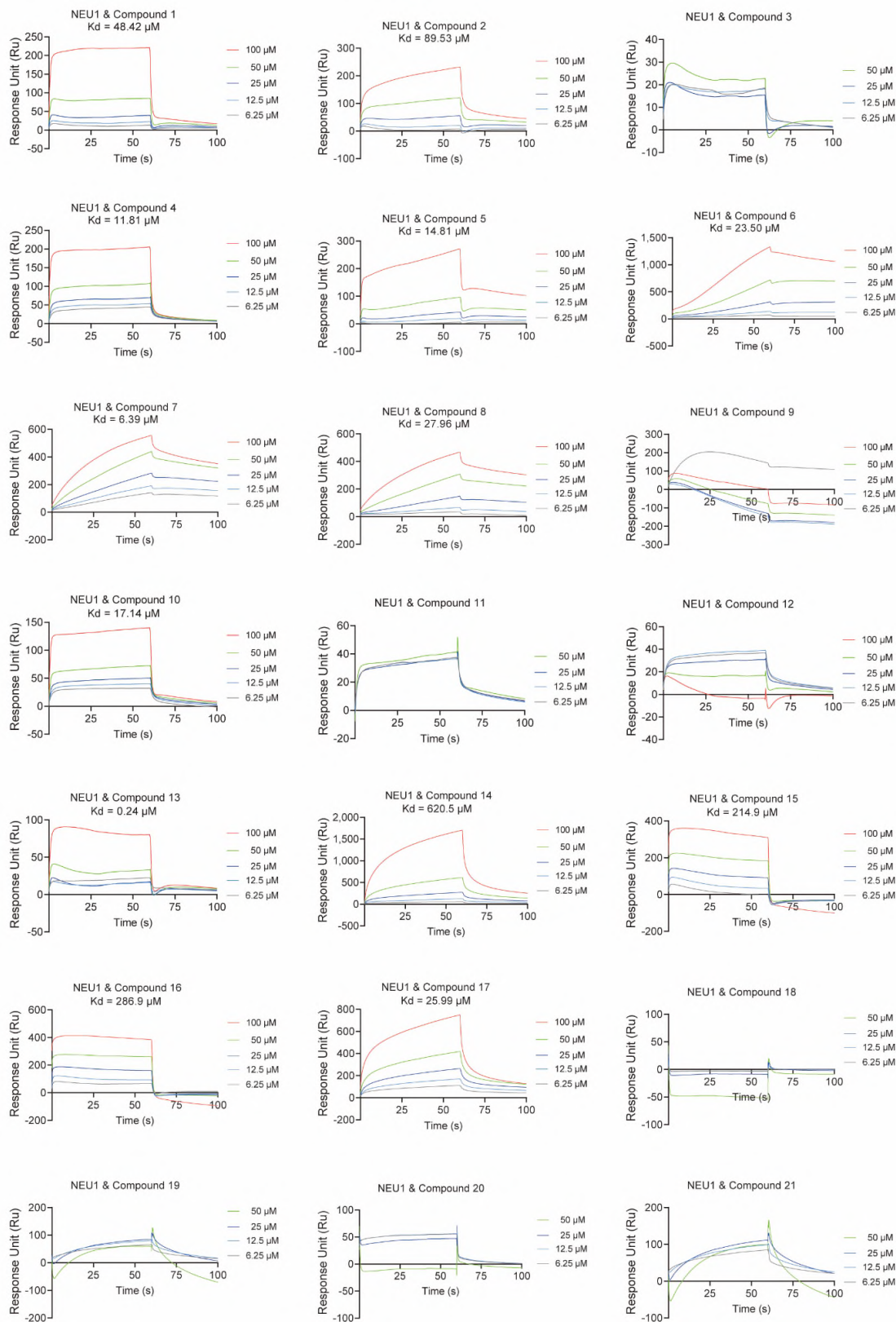

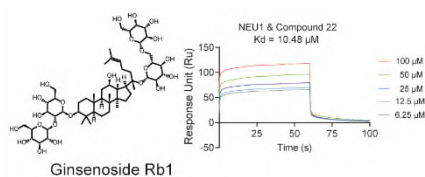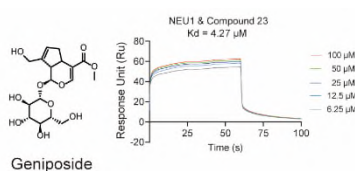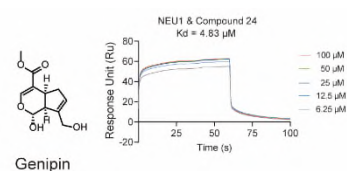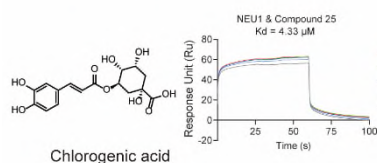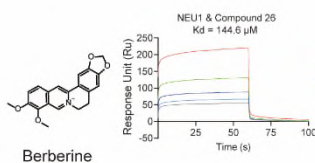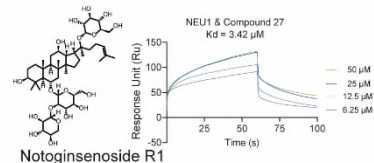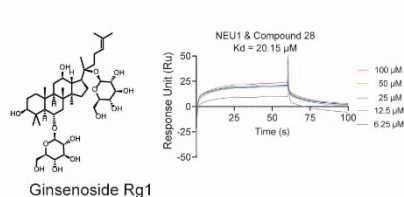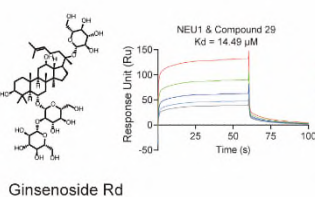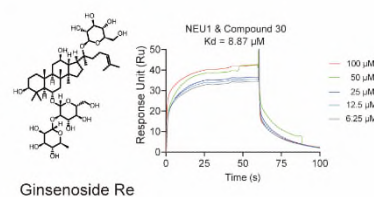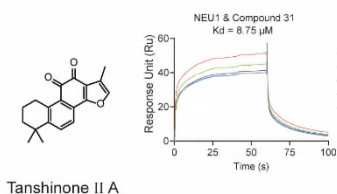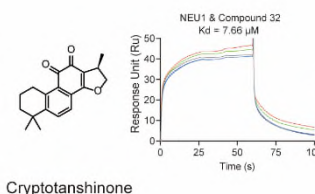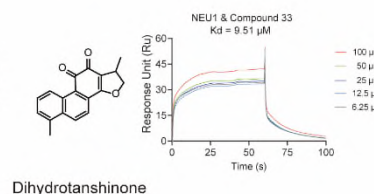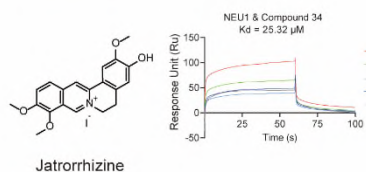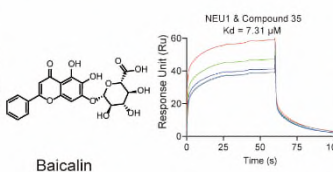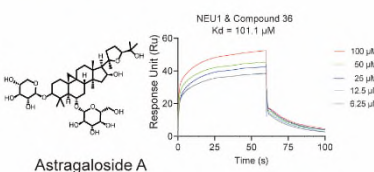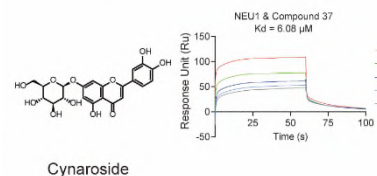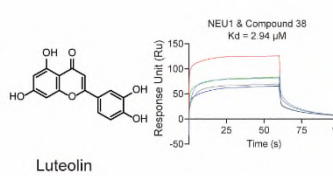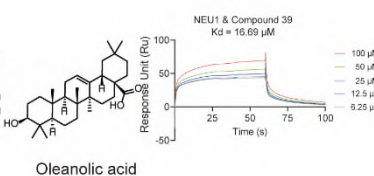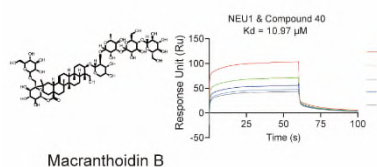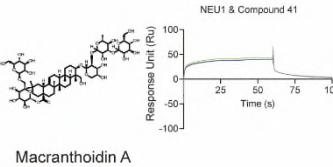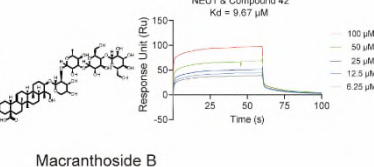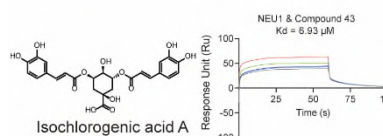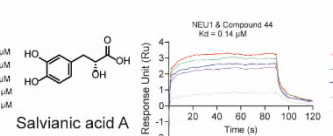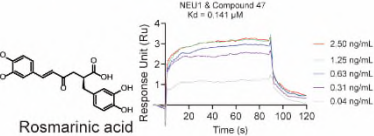



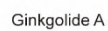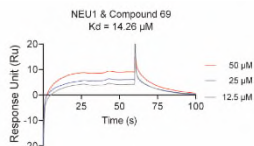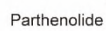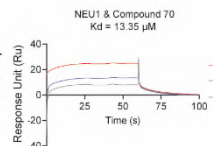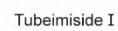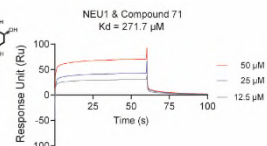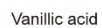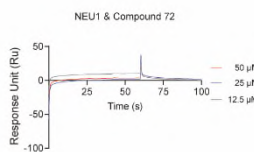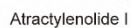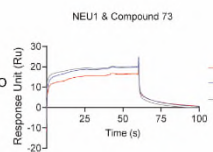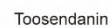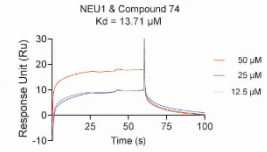

Supplement: Supplementary file 4 — Supplementary Data 2 [file 41467_2023_37450_MOESM4_ESM.pdf]
